# The Curli Accessory Protein CsgF of *Salmonella Typhimurium* Influences the in vitro Aggregation of Human Islet Amyloid Polypeptide

**DOI:** 10.17912/micropub.biology.001565

**Published:** 2025-05-19

**Authors:** Osmar Meza-Barajas, Clayton Connelly, Alejandra Lopez, Isamar Aranda, Ashwag Binmahfooz, Allison Newell, Sajith Jayasinghe

**Affiliations:** 1 Chemistry and Biochemistry, California State University, San Marcos, San Marcos, California, United States

## Abstract

The Curli secretion gene product F (CsgF) is a critical component of the assembly of Curli, proteinaceous filaments, found on the outer surface of gram-negative bacteria such as *E. Coli* and *Salmonella* . Herein we describe the ability of CsgF to influence the in-vitro aggregation of human islet amyloid polypeptide (hIAPP), an amyloidogenic polypeptide that is unrelated to Curli. In the presence of CsgF no increase in Thioflavin T fluorescence was observed for freshly solubilized hIAPP monitored as a function of time, suggesting that CsgF prevents the aggregation of hIAPP during the period of observation. A variant of CsgF lacking the first 65 residues in the N-terminus of CsgF retained the ability to inhibit the aggregation of hIAPP suggesting that the ability of CsgF to inhibit the aggregation of hIAPP is mediated by the C-terminal half of the protein.

**Figure 1. S. Typhimurium CsgF inhibits the aggregation of hIAPP:**
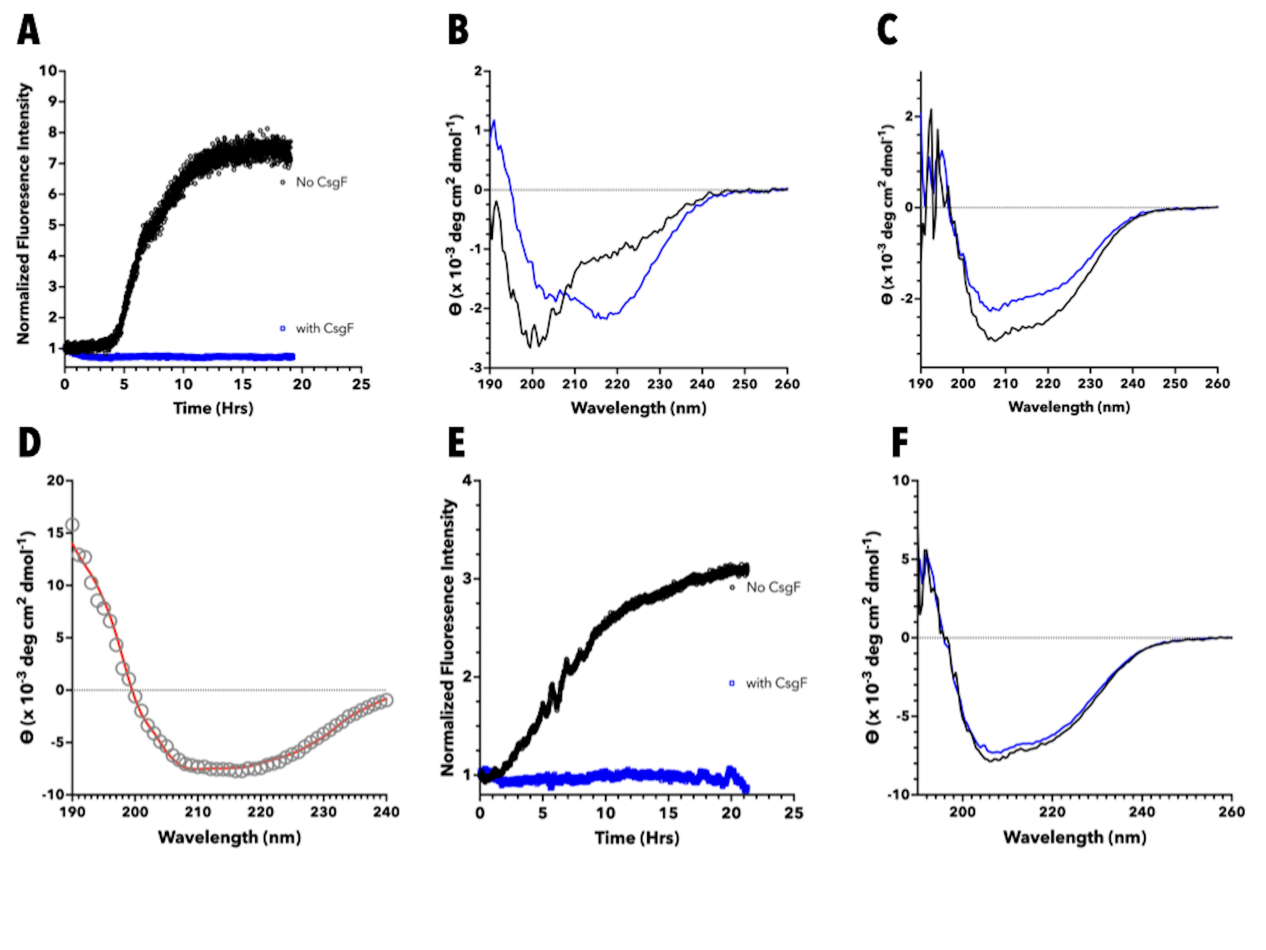
**(A)** Aggregation of 12.5 μM hIAPP in 20 mM phosphate buffer was monitored using ThT fluorescence intensity at 482 nm. In order to facilitate comparison between different samples ThT intensity immediately after mixing hIAPP with *S. Typhimurium* wild type CsgF (CsgF _ST-WT_ ) is normalized to 1. In order to be able to compare the differences in hIAPP aggregation in the absence and presence of CsgF _ST-WT_ one hIAPP sample was divided into two. Both hIAPP samples, one without CsgF _ST-WT_ , and the other with, were monitored simultaneously on two spectrofluorometers. In the absence of CsgF _ST-WT_ (black symbols) there is significant increase in ThT florescence intensity demonstrating hIAPP aggregation. In the presence of CsgF _ST-WT_ (blue symbols) no increase in ThT fluorescence intensity is observed indicating an absence of hIAPP aggregation. **(B)** CD spectra of 12.5 μM hIAPP (in the absence of CsgF _ST-WT_ ) immediately after rehydrating in buffer (black line) and at the end of the ThT time course (~ 20 hrs, blue line). The CD spectrum of hIAPP immediately after rehydrating in buffer is indicative of a predominantly unordered structure (negative ellipticity ~ 200 nm). The spectrum obtained at the end of the ThT time course contains negative intensity at ~ 218 nm and is indicative of the appearance of β-sheet secondary structure as expected for the aggregation of hIAPP. ( **C** ) CD spectra obtained immediately after mixing 12.5 μM CsgF _ST-WT_ with 12.5 μM hIAPP (black line) and at the end of the ThT time course (blue line). Although the spectrum at the end of the time course exhibits less negative intensity between 200-240 nm there does not appear to be any significant change in secondary structure. The ratio of intensity at 208 to 222 nm for the spectrum obtained at the end of the time course remains the same (1.28) as the ratio obtained from the spectrum immediately after mixing CsgF _ST-WT_ with hIAPP. **(D)** The CD spectrum of a variant of CsgF where the first 66 residues of the protein are removed (CsgF _Δ66_ ) in 20 mM phosphate buffer. The spectrum was deconvoluted using DICHROWEB and CsgF _Δ66_ was estimated to contain approximately 15 % α-helix, 30% β-strand, and 20% turn secondary structure distribution with 35% of the protein being unstructured. The fitted spectrum obtained via DICHROWEB (using the CONTIN method) is shown in red. **(E)** Aggregation of 12.5 μM hIAPP in 20 mM phosphate buffer monitored in the absence (black symbols) and presence (blue symbols) of CsgF _Δ66_ . No increase in ThT intensity was observed in the presence of CsgF _Δ66_ which suggests that the N-terminus of CsgF is not needed for the ability of the protein to inhibit the aggregation of hIAPP. **(F)** CD spectra obtained immediately after mixing 12.5 μM CsgF _Δ66_ with 12.5 μM hIAPP (black line) and at the end of the ThT time course (blue line) show no change in the overall secondary structure of the mixture consistent with a lack of aggregation of hIAPP. CD spectra in panels B, C, and F were obtained with minimal averaging (1-2 scans) to reduce the possibility of contributions from multiple conformations present during the aggregation process to the CD spectrum, and therefore contain some noise. The level of noise present in this data does not distract from the overall conclusions drawn from these data.

## Description

Curli, proteinaceous filaments found on the outer surface of bacteria such as *E. Coli* and *Salmonella* , are involved in biofilm formation and bacterial attachment to surfaces (Collinson et al., 1997) (Sukupolvi et al., 1997) (Austin et al., 1998) (Römling et al., 1998). Investigation of Curli has shown that these fimbriae possess characteristics similar to amyloid fibers found in a number of diseases such as Alzheimer’s disease, Parkinson’s disease, and type II diabetes. Similar to amyloid, Curli were found to change the spectral properties of Congo Red and Thioflavin T (ThT), as well as contain significant β-sheet character (Chapman et al., 2002). In contrast to amyloid found in disease, which are the result of protein misfolding and are toxic to cells, Curli has a functional role in bacteria. Given these observations Curli have been classified as a functional amyloid: a protein aggregate with amyloid like properties that also serves a functional role. Curli assembly involves six proteins, Csg A, B, C, E, F, and G (Chapman et al., 2002) . CsgA is the major protein component of Curli filaments while CsgB is thought to function as a nucleator of CsgA aggregation as well as to anchor CsgA to the outer surface of the bacterium (Hammar et al., 1996) (Bian & Normark, 1997) . CsgC, CsgE, CsgF, and CsgG provide a variety of important support functions. CsgC and CsgE, periplasmic proteins, prevent the intracellular aggregation of CsgA and/or CsgB (Gibson et al., 2007) (Taylor et al., 2011) (Evans et al., 2015). CsgF, found on the outer surface of bacteria, facilitates the extracellular assembly of CsgA into Curli. In the absence of CsgF, CsgA and CsgB do not localize to the cell surface and are secreted away (Chapman et al., 2002) (Robinson et al., 2006) (Nenninger et al., 2009). CsgG, an outer-membrane channel, secretes the extracellular proteins to the outside milieu (Cao et al., 2014) (Goyal et al., 2014).

The in vitro aggregation of CsgA, the major protein component of Curli, is inhibited by CsgF (Schubeis et al., 2018) and we sought to determine if CsgF is able to inhibit the aggregation of other amyloid forming proteins, and investigated its ability to influence the aggregation of human Islet Amyloid Polypeptide (hIAPP), a 37-residue polypeptide which aggregates to form amyloid deposits found in the pancreas of type II diabetics.

***Salmonella*** ***Typhimurium CsgF Inhibits The Aggregation of hIAPP*** : To determine the influence of CsgF on the aggregation of hIAPP the real time fluorescence intensity of ThT at 482 nm was measured in the absence and presence of *S. typhimurium* wild type CsgF (CsgF _ST-WT_ ). In the absence of CsgF _ST-WT_ freshly prepared hIAPP exhibited a sigmoidal increase in ThT intensity ( Figure 1A, black symbols). We also monitored the secondary structure of hIAPP immediately after initiating aggregation and at the end of the ThT time course. The circular dichroism (CD) spectrum of freshly dissolved hIAPP exhibited a peak with negative ellipticity at ~ 198 nm suggestive of a predominantly unstructured backbone ( Figure 1B, black line), while the spectrum obtained after the increase in ThT intensity contained a peak with negative ellipticity at ~ 218 nm, suggestive of a predominantly β-sheet backbone structure ( Figure 1B, blue line). These observations are similar to those described in the literature for hIAPP aggregation (Kapurniotu, 2001) (Padrick & Miranker, 2002) (Jayasinghe & Langen, 2004) . Incubating hIAPP with CsgF _ST-WT_ at a 1:1 mole ratio completely abolished the increase in ThT intensity ( Figure 1A, blue symbols). We confirmed the impact of CsgF _ST-WT_ on the aggregation of hIAPP using five independent measurements (see extended data). For each experiment a single solution of hIAPP was separated into two cuvettes and monitored simultaneously with ThT in the absence and presence of CsgF _ST-WT_ . This allowed us to be more confident that any impact we observed with CsgF was due to the protein itself and not because the hIAPP preparation did not aggregate. We also monitored structural changes using CD spectroscopy. Unlike in the case of hIAPP alone no significant change was observed between the CD spectrum obtained immediately after mixing CsgF _ST-WT_ with hIAPP and the spectrum obtained at the end of the ThT time course ( Figure 1C ). Although it is difficult to attribute structural changes to a specific polypeptide contained in a mixture, we expect any conversion of hIAPP from a predominantly random coil to a 𝛽-sheet structure to manifest as a reduction in the negative ellipticity at ~ 200 nm and an increase in negative ellipticity at ~ 218 nm. The ratio of intensities at 208 and 222 nm are 1.28 for the CD spectra obtained immediately after mixing hIAPP with CsgF _ST-WT_ and at the end of the observation period, suggesting the absence of any significant secondary structure change during the period of observation. Taken as a whole these data suggest that CsgF is able to inhibit the aggregation of hIAPP.

***The N-terminal residues of* CsgF _ST-WT_ *are not needed to inhibit the aggregation of hIAPP*** : CsgF _ST-WT_ is a predominantly unstructured protein (Green et al., 2016). The solution structure of *E. Coli* CsgF indicates that the entire N-terminal half of the protein is unstructured while the C-terminal half of the protein contains an 𝛼-helix followed by a 𝛽-sheet comprised of four antiparallel 𝛽-strands (Schubeis et al., 2018). It has been speculated that the N-terminus may contain an export signal (Schubeis et al., 2018), and the structure of the Curli secretion channel CsgG in complex with CsgF suggests that the N-terminus of CsgF interacts with CsgG via 27 N-terminal residues (Zhang et al., 2020). We analyzed the CD spectrum of a variant of CsgF _ST-WT_ lacking the first 66 residues of CsgF (CsgF _Δ66_ ) using DichroWeb (Whitmore & Wallace, 2004, 2008) and found a secondary structure distribution of approximately 15 % α-helix, 30% β-strand, 20% turn, with 35% of the protein being unstructured ( Figure 1D ). This distribution compares favorably with the secondary structure distribution calculated fron the solution NMR structure of *E. Coli* CsgF (approximately 21% 𝛼-helix, 38% 𝛽-strand, 11% turn, and 29% unstructured), suggesting that removal of the N-terminal unstructured region does not alter the overall secondary structure distribution of the remaining amino acid sequence. To determine if the N-terminal region of CsgF plays a role in the protein’s ability to inhibit the aggregation of hIAPP, aggregation was monitored in the presence of CsgF _Δ66_ . Freshly rehydrated hIAPP incubated in the presence of CsgF _Δ66_ ( Figure 1E, blue symbols) did not exhibit any increase in ThT intensity at 482 nm. We repeated these experiments with five independent preparations of hIAPP and in each case observed no increase in ThT intensity when hIAPP was incubated with CsgF _Δ66_ (see extended data). We also monitored changes in secondary structure. Again, no significant change was observed between the CD spectrum obtained immediately after mixing CsgF _Δ66_ with hIAPP and the spectrum obtained at the end of the ThT timecourse ( Figure 1F ), which is consistent with a lack of aggregation of hIAPP. Thus, it appears that CsgFD66 is still able to inhibit the aggregation of hIAPP, suggesting that the N-terminal unstructured region of CsgF is not crucial to this activity.

Although intriguing, the ability of a Curli accessory protein to inhibit the aggregation of an amyloidogenic protein unrelated to Curli formation is not unique. ThT aggregation studies have shown that CsgC is able to prevent the in vitro aggregation of human α-synuclein, an amyloidogenic protein implicated in Parkinson’s disease (Evans et al., 2015). The ability of CsgC to prevent protein aggregation appears to be specific since it was unable to prevent the aggregation of the Alzheimer’s Aβ _42_ peptide even at a 1:1 (CsgC:Aβ _42_ ) molar ratio. Based on a comparison of the primary structure of CsgA and α-synuclein, it has been suggested that these two proteins interact with CsgC via a common interaction motif (Evans et al., 2015) . In contrast, there is no discernible region of sequence similarity between CsgA and hIAPP, suggesting that the ability of CsgF _ST-WT_ to inhibit the aggregation of hIAPP does not involve the recognition of a specific amino acid sequence.

**Conclusion:** The observation that CsgF _ST-WT_ can influence the aggregation of hIAPP adds to the growing body of evidence indicating that the Curli accessory proteins CsgC, CsgE, and CsgF can modulate the aggregation of proteins that form amyloid. Future studies aimed at more clearly identifying the regions and residues of CsgF involved in interacting with hIAPP could help our understanding of the mechanism by which CsgF inhibits protein aggregation.

## Methods

***Expression and purification of C-terminal hexahistidine tagged Salmonella Typhimurium* *CsgF:*** *E. Coli* BL21 (DE3) cells transformed with the appropriate pET21 vector containing the sequence for *Salmonella Typhimurium* CsgF (wild type or lacking the first 66 residues), fused to the plasmid encoded C-terminal hexahistidine tag, were grown, at 37 ^0^ C to a OD (595 nm) of between 0.5 - 1. Protein production was induced with the addition of 1 mM IPTG, and the cells harvested by centrifugation after 16 hours of incubation at 26 ^0^ C. Cells were resuspended in binding buffer (20 mM phosphate, 20 mM imidazole, 500 mM NaCl ) and B-PER, frozen using liquid nitrogen and lysed using manual grinding with a mortar and pestle. Unbroken cells, and cell debris, were removed by centrifugation (5000 x g) for 20 min at 4 ^0^ C, and histidine tagged protein was recovered from the lysate using a HiTrap TALON Crude Co ^2+^ column (GE Healthcare, Piscataway, NJ) as described by the manufacturer. Resin bound protein was eluted from the column using elution buffer (20 mM phosphate, 250 mM imidazole, 500 mM NaCl) and subsequently desalted and buffer exchanged (in to 20 mM phosphate buffer) using a HiPrep 26/10 Desalting column (GE Healthcare, Piscataway, NJ). The purity of affinity purified histidine tagged CsgF was investigated using SDS PAGE and the presence of the histidine tag was confirmed by western blot using an Anti-His (C-Term)-HRP antibody (LifeTechnologies, Carlsbad, CA).

***Preparation of hIAPP* :** Lyophilized human IAPP peptide was dissolved in Hexafluoro Isopropanol (HFIP) to obtain clear solutions. Peptide concentrations were calculated and aliquots of peptide in HFIP were pipetted into 1.5 mL Eppendorf tubes, mixed with 500 μL of deionized distilled water, immediately frozen in liquid nitrogen, and lyophilized overnight. Dry lyophilized peptide was stored in a vacuum desiccator until use.

***Preparation of hIAPP samples to monitor aggregation:*** Dry lyophilized peptide prepared as described above was dissolved in 20 mM buffer at pH 7.4. The solution was separated into two samples of equal volume. To one CsgF or CsgFD66 was added from a stock solution while an equal volume of buffer was added to the other. The final concentration of hIAPP in each sample was 12.5 or 25 μM.

***ThT fluorescence assay for protein aggregation:*** Aggregation of hIAPP was monitored using the fluorescence intensity increase of ThT. To each aggregation reaction (prepared as described above), a sufficient amount of ThT to yield 25 μM (from 5 mM stock solution in deionized distilled water) was added immediately after dissolving hIAPP in buffer, and real-time emission intensities were measured at 482 nm with excitation at 450 nm. Measurements were performed at room temperature with excitation and emission slit widths of 1 and 10 nm, respectively. Fluorescence measurements were taken using a FluoroMax-3 Spectro-fluorometer (Horiba Jobin Yvon Inc, Edison, NJ).

***CD Spectroscopy:*** CD spectra were obtained using a Jaco 810 spectropolarimeter (Jasco Inc., Easton, MD). Measurements were taken every 0.5 nm at a scan rate of 50 nm/min with an averaging time of 1 s. All spectra were collected between 190 and 260 nm using a 2 mm path length quartz cuvette. Spectra were corrected by subtracting an appropriate background, and are presented with intensity units of molar ellipticity.

## Reagents

pET21 vectors containing the inserted sequences for CsgF fused to the plasmid-encoded C-terminal hexahistidine tag were obtained from Genscript (Piscataway, NJ). *E. Coli* BL21(DE) expression competent cells, Bacterial Protein Extraction Reagent (B-PER), and a C-terminal AntiHis antibody were obtained from ThermoFischer Scientific (Grand Island, NY). Hexafluoroisopropanol (HFIP) and ThT were obtained from Sigma-Aldrich (Milwaukee, WI). Synthetic wild-type human Islet Amyloid Polypeptide (h-IAPP) was obtained from Bachem (King of Prussia, PA) or from PolyPeptide Laboratories (Torrence, CA).

## Data Availability

Description: ThT Fluorescence time course data for hIAPP in the absence and presence of wild type CsgF and variant lacking N-terminal 66 residues. Resource Type: Image. DOI: https://doi.org/10.22002/a67hh-s2s51

## References

[R1] Austin John W, Sanders Greg, Kay William W, Collinson S.Karen (1998). Thin aggregative fimbriae enhance
*Salmonella enteritidis*
biofilm formation. FEMS Microbiology Letters.

[R2] Bian Zhao, Normark Staffan (1997). Nucleator function of CsgB for the assembly of adhesive surface organelles in Escherichia coli. The EMBO Journal.

[R3] Cao Baohua, Zhao Yan, Kou Yongjun, Ni Dongchun, Zhang Xuejun Cai, Huang Yihua (2014). Structure of the nonameric bacterial amyloid secretion channel. Proceedings of the National Academy of Sciences.

[R4] Chapman Matthew R., Robinson Lloyd S., Pinkner Jerome S., Roth Robyn, Heuser John, Hammar Mårten, Normark Staffan, Hultgren Scott J. (2002). Role of
*Escherichia coli*
Curli Operons in Directing Amyloid Fiber Formation. Science.

[R5] Collinson S. Karen, Clouthier Sharon C., Doran James L., Banser Pamela A., Kay William W. (1997). Characterization of the AgfBA Fimbrial Operon Encoding Thin Aggregative Fimbriae of Salmonella Enteritidis. Advances in Experimental Medicine and Biology.

[R6] Evans Margery L., Chorell Erik, Taylor Jonathan D., Åden Jörgen, Götheson Anna, Li Fei, Koch Marion, Sefer Lea, Matthews Steve J., Wittung-Stafshede Pernilla, Almqvist Fredrik, Chapman Matthew R. (2015). The Bacterial Curli System Possesses a Potent and Selective Inhibitor of Amyloid Formation. Molecular Cell.

[R7] Gibson D. L, White A. P, Rajotte C. M, Kay W. W (2007). AgfC and AgfE facilitate extracellular thin aggregative fimbriae synthesis in Salmonella Enteritidis. Microbiology.

[R8] Goyal Parveen, Krasteva Petya V., Van Gerven Nani, Gubellini Francesca, Van den Broeck Imke, Troupiotis-Tsaïlaki Anastassia, Jonckheere Wim, Péhau-Arnaudet Gérard, Pinkner Jerome S., Chapman Matthew R., Hultgren Scott J., Howorka Stefan, Fronzes Rémi, Remaut Han (2014). Structural and mechanistic insights into the bacterial amyloid secretion channel CsgG. Nature.

[R9] Green Amanda, Pham Nguyen, Osby Krystle, Aram Alexander, Claudius Rochelle, Patray Sharon, Jayasinghe Sajith A. (2016). Are the curli proteins CsgE and CsgF intrinsically disordered?. Intrinsically Disordered Proteins.

[R10] Hammar M, Bian Z, Normark S (1996). Nucleator-dependent intercellular assembly of adhesive curli organelles in Escherichia coli.. Proceedings of the National Academy of Sciences.

[R11] Jayasinghe Sajith A., Langen Ralf (2004). Identifying Structural Features of Fibrillar Islet Amyloid Polypeptide Using Site-directed Spin Labeling. Journal of Biological Chemistry.

[R12] Kapurniotu Aphrodite (2001). Amyloidogenicity and cytotoxicity of islet amyloid polypeptide. Biopolymers.

[R13] Nenninger Ashley A., Robinson Lloyd S., Hultgren Scott J. (2009). Localized and efficient curli nucleation requires the chaperone-like amyloid assembly protein CsgF. Proceedings of the National Academy of Sciences.

[R14] Padrick Shae B., Miranker Andrew D. (2002). Islet Amyloid:  Phase Partitioning and Secondary Nucleation Are Central to the Mechanism of Fibrillogenesis. Biochemistry.

[R15] Robinson Lloyd S., Ashman Elisabeth M., Hultgren Scott J., Chapman Matthew R. (2005). Secretion of curli fibre subunits is mediated by the outer membrane‐localized CsgG protein. Molecular Microbiology.

[R16] Römling Ute, Bian Zhao, Hammar Mårten, Sierralta Walter D., Normark Staffan (1998). Curli Fibers Are Highly Conserved between
*Salmonella typhimurium*
and
*Escherichia coli*
with Respect to Operon Structure and Regulation. Journal of Bacteriology.

[R17] Schubeis Tobias, Spehr Johannes, Viereck Janika, Köpping Laura, Nagaraj Madhu, Ahmed Mumdooh, Ritter Christiane (2018). Structural and functional characterization of the Curli adaptor protein CsgF. FEBS Letters.

[R18] Sukupolvi S, Lorenz R G, Gordon J I, Bian Z, Pfeifer J D, Normark S J, Rhen M (1997). Expression of thin aggregative fimbriae promotes interaction of Salmonella typhimurium SR-11 with mouse small intestinal epithelial cells. Infection and Immunity.

[R19] Taylor Jonathan D., Zhou Yizhou, Salgado Paula S., Patwardhan Ardan, McGuffie Matt, Pape Tillmann, Grabe Grzegorz, Ashman Elisabeth, Constable Sean C., Simpson Peter J., Lee Wei-chao, Cota Ernesto, Chapman Matthew R., Matthews Steve J. (2011). Atomic Resolution Insights into Curli Fiber Biogenesis. Structure.

[R20] Whitmore L., Wallace B. A. (2004). DICHROWEB, an online server for protein secondary structure analyses from circular dichroism spectroscopic data. Nucleic Acids Research.

[R21] Whitmore Lee, Wallace B. A. (2007). Protein secondary structure analyses from circular dichroism spectroscopy: Methods and reference databases. Biopolymers.

[R22] Zhang Manfeng, Shi Huigang, Zhang Xuemei, Zhang Xinzheng, Huang Yihua (2020). Cryo-EM structure of the nonameric CsgG-CsgF complex and its implications for controlling curli biogenesis in Enterobacteriaceae. PLOS Biology.

